# SSRI Treatment Response Prediction in Depression Based on Brain Activation by Emotional Stimuli

**DOI:** 10.3389/fpsyt.2020.538393

**Published:** 2020-11-13

**Authors:** Antonia Preuss, Bianca Bolliger, Wenzel Schicho, Josef Hättenschwiler, Erich Seifritz, Annette Beatrix Brühl, Uwe Herwig

**Affiliations:** ^1^Department of Psychiatry, Psychotherapy and Psychosomatics, University Hospital for Psychiatry, Zurich, Switzerland; ^2^Clinic for Psychiatry and Psychotherapy Clienia, Oetwil am See, Switzerland; ^3^Center for Treatment of Anxiety and Affection Disorder Zentrum für Angst- und Depressionsbehandlung Zürich (ZADZ), Zurich, Switzerland; ^4^Center for Psychiatry Reichenau, Academic Hospital University of Konstanz, Konstanz, Germany

**Keywords:** major depressive disorder (MDD), functional magnet resonance imaging (fMRI), emotional stimuli, treatment-outcome, SSRI (selective serotonin reuptake inhibitor)

## Abstract

**Introduction:** The prediction of antidepressant treatment response may improve outcome. Functional magnetic resonance imaging (fMRI) of emotion processing in major depressive disorder (MDD) may reveal regional brain function serving as predictors of response to treatment with selective serotonin reuptake inhibitor (SSRI).

**Methods:** We examined the association between pre-treatment neural activity by means of fMRI during the perception of emotional stimuli in 22 patients with MDD and the treatment outcome after 6 weeks' medication with an SSRI. A whole brain correlation analysis with Beck Depression Inventory (BDI) change between pre- to post-treatment was conducted to identify neural regions associated with treatment response.

**Results:** During the perception of positive stimuli, responders were characterized by *more activation* in posterior cingulate cortex (PCC), medial prefrontal cortex, and thalamus as well as middle temporal gyrus. During perception of negative stimuli, PCC, and pregenual anterior cingulate cortex showed the highest correlation with treatment response. Furthermore, responders exhibited *higher activation* to emotional stimuli than to neutral stimuli in all the above-mentioned regions, while non-responders demonstrated an attenuated neural response to emotional compared to neutral stimuli.

**Conclusion:** Our data suggest that the activity of distinct brain regions is correlated with SSRI treatment outcome and may serve as treatment response predictor. While some regions, in which activity was correlated with treatment response, can be assigned to networks that have been implied in the pathophysiology of depression, most of our regions of interest could also be matched to the default mode network (DMN). Higher DMN activity has been associated with increased rumination as well as negative self-referential processing in previous studies. This may suggest our responders to SSRI to be characterized by such dysregulations and that SSRIs might modify the function associated with this network.

## Introduction

Clinically useful predictors of antidepressant treatment outcome in major depressive disorder (MDD) are needed. While antidepressants have been demonstrated efficient in treating MDD, there are differences in individual response (1, 2). Also, due to the time needed to take effect, every non-response with change of medication means weeks of continued depression (3).

Altered neural responses in psychiatric illnesses and neural modulation of psychopharmacological drugs have been investigated for years (4–9). Following, there has been an increasing amount of studies providing evidence that neural activity could function as a biomarker for response to different treatment approaches (10–12). For example, activity in the anterior cingulate cortex was found to predict treatment response (13). Negative cognitive biases, e.g., a bias toward the perception of negative rather than positive stimuli in MDD are generally accepted alterations in depression (14), with several models seeking to explain the underlying alterations on a neural level (7, 15, 16). Negatively biased thoughts and maladaptive rumination in depression has also been linked with heightened activity of the default mode network (DMN) (17). The DMN refers to a network which was originally thought to be active only during resting state (18), but research shows its function to go beyond that (19) and disruption of DMN function in psychiatric illnesses (20, 21). In a newer model of antidepressant drug action, they have been suggested to remediate negative affective biases by changing the processing of emotionally valenced information (22). Such negative biases have been studied by our research group, with (23) showing a neural correlate of a pessimistic attitude in healthy participants during expectation of unpleasant stimuli and stimuli of unknown valence. A similar setup in depressed patients identified distinct regions potentially corresponding to a negative cognitive bias in MDD (24, 25). Based on these results, we hypothesized a difference in information processing when perceiving/expecting emotional stimuli in MDD depending on improvement by treatment with a selective serotonin reuptake inhibitor (SSRI) for 6 weeks.

## Materials and Methods

### Subjects

Thirty participants with MDD were recruited from the Psychiatric University Hospital in Zurich, its outpatient facility and the center for the treatment of anxiety and depression in Zurich (ZADZ). Inclusion criteria were moderate to severe MDD (Beckmann Depression Index, BDI ≥18 pt and/or Hamilton Rating Scale for Depression, HAM-D≥ 20) and righthandedness with no current pharmacological treatment. The decision for antidepressant therapy was not influenced by participating in the study, instead we recruited participants for which the beginning with antidepressant medication, especially SSRI, had already been planned.

The presence of MDD based on the Diagnostic and Statistical Manual of Mental Disorders (DSM-IV) was confirmed through a structured interview led by an experienced psychiatrist [Mini Neuropsychiatric Interview (26)]. Also the participants were asked to fill out the Beck Depression Inventory [BDI, (27)].

Participants were screened for exclusion criteria consisting of substance abuse, current antidepressant medication, psychiatric, or neurological comorbidities, previous head injuries as well as contraindications for magnet resonance imaging. Furthermore, pregnancy was excluded in female participants. This initial assessment took place within 2 days before the fMRI (functional magnetic resonance imaging) scan.

A total of eight participants had to be excluded: three due to technical problems with the fMRI which resulted in unusable material, one due to the diagnosis of a cerebral cyst, one participant decided against medication after undergoing the scan, two participants received only non-SSRI antidepressants and in one case follow up was not possible. The included participants received an SSRI for 6 weeks, after which the final assessment was done.

All participants gave informed consent after having received complete information about the study prior to inclusion. The study was approved by the local ethics committee in Zurich and was conducted per the ethical standards of the Declaration from Helsinki.

### Neuroimaging Task

**Figure 1:**
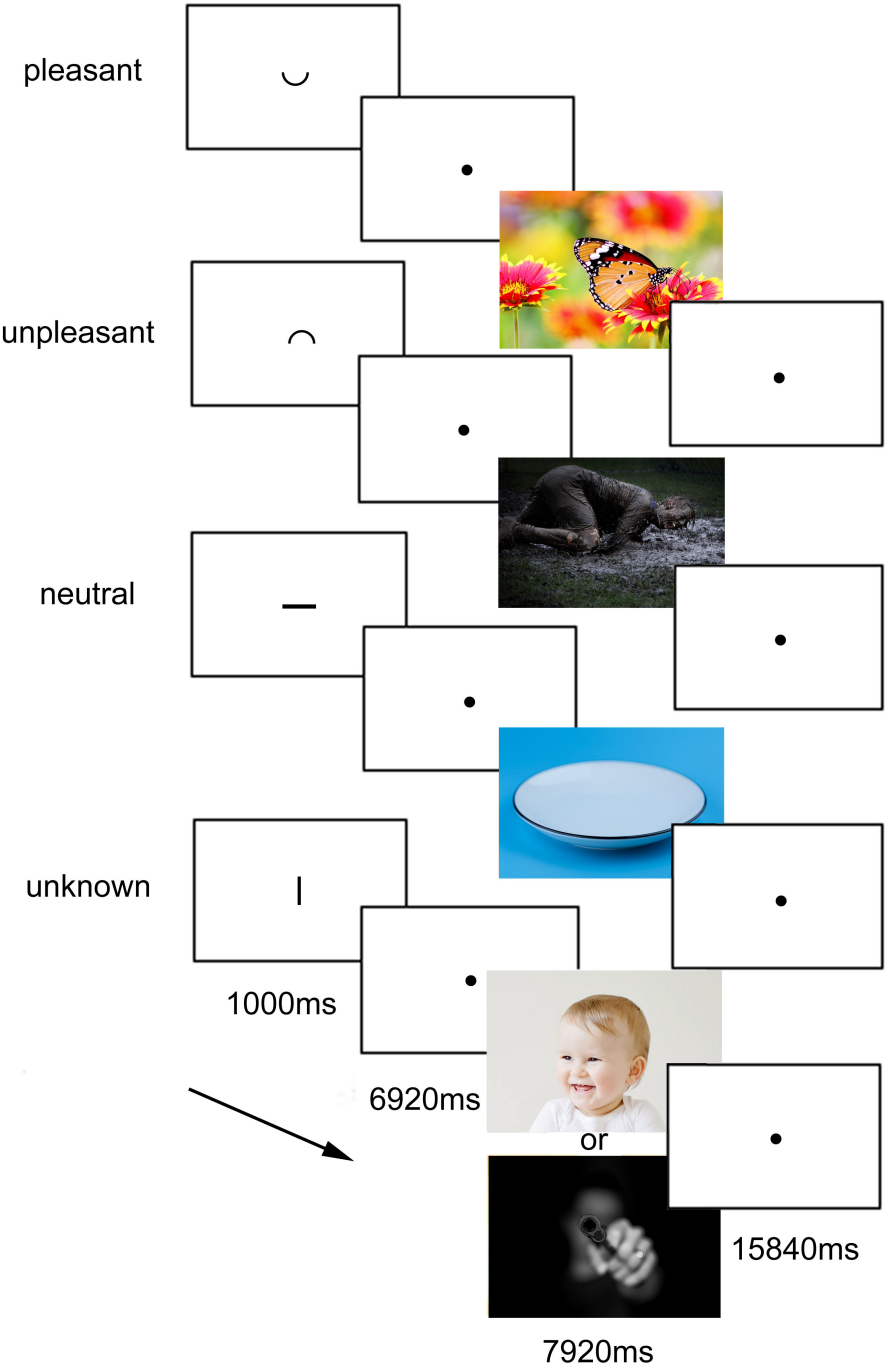
Experimental task. The four conditions with the respective cues and the duration are presented. The cues, presented for 1,000 ms, indicated the valence of the following picture, appearing after a delay of further 6,920 ms. In the figure, the cues are relatively enlarged for presentation reasons. In the experiment, they were about 1/40 of screen height. The experimental task design was identical to the one used and published before by Herwig et al. (24). The images used in the task came from the IAPS (International affective picture system), for which publication is not permitted, so for illustrative purposes here pictures under CC0 license are displayed.

While undergoing fMRI scanning, the participants completed a task consisting of 56 trials (programmed with Presentation, Neurobehavioral Systems, USA), in which they were presented cues pointing to the emotional valence of the following pictures. During each trial, the participants were presented the cue for a duration of 1,000 ms (in the size of 1/20 of the screen) which was either “ ⌣ ” for positive pictures, “  ⌢  ” for negative pictures, “—“for neutral pictures, or “|” for “unknown” pictures, which could be either positive or negative (see Figure 1). After the cue followed an anticipatory period of 6,920 ms, during which a fixation cross was displayed. The respective, screen-filling picture was shown for 7,920 ms = another 4 TR (repetition time), followed by a baseline of 15,840 ms = 8TR to allow blood oxygenation level dependent (BOLD) Levels to return to normal. Pictures from the International Affective Picture System (IAPS) were used.

The “unknown” condition was primarily for analysis of activation during the anticipatory period, the activation during the subsequently following negative or positive picture was not analyzed. Each of the four conditions consisted of 14 trials, which were displayed in randomized order. The whole neuroimaging task had a duration of about 30 min.

Participants were instructed about the task, to be aware about the emotional valence and to expect emotional pictures following the cues. The task was identical to the one used by Herwig et al. (24).

### Questionnaires

Additionally to the self-rating instrument BDI, a psychiatrist evaluated the scores for the Hamilton Rating Scale for Depression (HAM-D, (28) and the Montgomery–Åsberg Depression Rating Scale [MADRS, (29)], and the participants also completed a handedness questionnaire (Annett Hand Preference Questionnaire, and the Waterloo Handedness Questionnaire by Bryden) before undergoing the scan. Immediately afterwards, the participants rated the emotional valence of the presented pictures during the scan on a nine-point rating scale (shown again as printouts, 1—very negative, 9—very positive).

Furthermore, within a day after the scan, they were asked to fill out German versions of questionnaires concerning emotion regulation [Emotion Regulation Questionnaire, ERQ, (30)], personality traits [Eysenck Personality Inventory, EPI, (31)], and childhood traumatic experiences [Childhood Trauma Questionnaire, CTQ, (32)]. In Follow-ups after 1, 3, 5, and finally 6 weeks the medication status was assessed, HAM-D/MADRAS were re-evaluated by the same psychiatrist that did the initial assessment, and the participants asked again to complete the BDI via online-questionnaire.

### Image Acquisition

Imaging was performed with a 3.0T Philips Achieva Scanner (Philips Medical Systems, Best, The Netherlands, equipped with an 8-channel receive head-coil array). Echoplanar imaging was performed for functional MR imaging [repetition time (TR)/echo-time 1,980/60 ms, 33 sequential axial slices, whole brain, slice thickness 3.0 mm, field of view (FOV ap, fh, rl): 240 × 99 × 240 mm, matrix 80 × 80 voxel, resulting voxel size: 3 × 3 × 3 mm, axial orientation]. High-resolution 3-D T1 weighted anatomical volumes were acquired [TR/echo-time 600/20 ms, 145 slices, whole brain, slice thickness 1.2 mm, FOV 225 × 230 × 174 mm, matrix 224 × 224, voxel size 1 × 1 × 1 mm) for co-registration with the functional data. Also, T2-weighted images were acquired to enhance detection of brain abnormalities which would have led to exclusion [TR/echo-time 3,000/125 ms, 24 slices, whole brain, slice thickness 4 mm, FOV 230 × 119 × 184 mm, matrix 512 × 512 voxel, voxel size 0.5 × 0.5 × 4 mm). Stimuli were presented via digital goggles (Resonance Technologies, Northridge, CA, USA).

### MRI Data Analysis

fMRI data were analyzed using BrainVoyagerQX 2.8 (Brain Innovation, The Netherlands). Pre-processing included motion correction (using trilinear/sinc interpolation), slice scan time correction (using cubic spline interpolation), temporal high-pass filtering (using a GLM approach with 3 cycles), and removal of linear trends. Functional and 3-D structural measurements were co-registered and structural and functional datasets were transformed into Talairach space, resulting in a voxel size of 3 × 3 × 3 mm. Finally, the datasets were spatially smoothed with an 8-mm full-width half-maximum Gaussian kernel for subsequent group analysis. Eight predictors were used to build the design matrix, consisting of the anticipation and presentation conditions (respectively, positive, negative, neutral, and unknown).

The functional data were convoluted with a two-parameter gamma hemodynamic response function (HRF), provided by BrainVoyagerQX.

In the first stage of the analysis, we performed a random effects analysis in BrainVoyagerQX with separate subject predictors for each of the contrasts of interest (positive >neutral, negative >neutral, expectation negative >exp. neutral, exp. unknown >exp. neutral). In the next step, separate beta maps for each subject were computed, and correlation on the whole brain level with the change in BDI (pre-treatment BDI—post-treatment BDI) was conducted to identify regions of interest, separately for each contrast. The statistical significance level was set at *p* < 0.005 or lower. As a multiple comparisons correction, the cluster-level statistical threshold estimator was used with 1,000 iterations to calculate the minimal cluster size, provided as plugin in BrainvoyagerQX (Monte Carlo Simulation). This resulted in our primary outcome, various clusters which we defined as regions of interest (ROI), of which the beta weights were extracted for further analysis and external validation of the result. Identification of anatomical regions was based on the Talairach system. We also performed exploratory analysis of the anticipation and presentation (contrast exp. positive >exp. neutral, which was not previously defined as contrast of interest), and which yielded no results of interest.

### Further Statistical Analysis

The beta weights from the correlation analysis were visualized using scatter plots.

The results from the neuroticism and extraversion score from the personality inventory, ERQ, and CTQ results were correlated with depression scores (pre-treatment BDI as well as change in BDI) and beta weights of regions of interest using Spearman's Correlation. The average and standard deviation in picture rating was also correlated with depression scores, as well as change of BDI during treatment, using Spearman's Correlation as well. To allow for a group comparison regarding the results of the questionnaires as well as clinical factors, participants were divided into responders (Reduction of BDI >=50%) and non-responders (Reduction of BDI <50%).

We conducted an independent samples *t*-test between responders and non-responders for the questionnaires to search for significant differences, also we applied a chi-square test to seek differences in the two groups regarding various clinical factors.

Statistical analysis was conducted using SPSS (IBM SPSS Statistics, Version 24).

## Results

### Epidemiological and Behavioral Analysis

The 22 included participants had an average age of 39.5 y (range 20–63 y, standard deviation 11.5 y), 9 were male, 13 female. Mean pre-treatment BDI was 27 pts (range 17–41, mean responders 30 pts, mean non-responders 25.2 pts), mean post-treatment BDI was 18.4 pts (range 3–55 pts, mean responders 8.4 pts, mean non-responders 24.1 pts) (see Table 1, Supplementary Table 1). The analysis of the depression questionnaires (BDI, HAMD, MADRS) at T3 and T5 showed no unexpected developments or outliers, so the main analysis was done with the values of T1 and T6. The division between the two groups (responders and non-responders) was relatively clear, with the “best” non-responder having a change of 35% in BDI, and the responder with the least improvement having a change in BDI of 58%.

**Table 1:** Demographics, questionnaires and clinical factors of participants.

	**All**			**Responder**			**Non-responder**		
	***n***		***S.D*.**	***n***		***S.D*.**	***n***		***S.D*.**
*n*		**22.0**			**8.0**			**14.0**	
Mean age, years		**39.5**	*11.5*		**38.0**	*14.6*		**40.3**	*9.9*
**Gender**									
Male		**9.0**			**5.0**			**4.0**	
Female		**13.0**			**3.0**			**10.0**	
**Mean depression and anxiety scores**									
BDI T1	*22*	**27.0**	*8.0*	*8*	**30.0**	*7.3*	*14*	**25.2**	*8.2*
BDI T6	*22*	**18.4**	*12.0*	*8*	**8.5**	*4.3*	*14*	**24.1**	*11.2*
HAM-D T1	*22*	**25.5**	*6.1*	*8*	**26.3**	*4.5*	*14*	**25**	*6.9*
HAM-D T6	*22*	**18.1**	*10.1*	*8*	**8.3**	*5.7*	*14*	**23.6**	*9.1*
MADRS T1	*22*	**28.6**	*7.4*	*8*	**30.5**	*7.3*	*14*	**27.6**	*7.6*
MADRS T6	*21*	**20.4**	*10.1*	*7*	**9.9**	*3.7*	*14*	**25.7**	*7.7*
**Chronology and clinical factors**									
First episode of MDD	*16*	**72.7%**		*5*	**62.5%**		*11*	**78.6%**	
Recurring MDD (average previous episodes)	*6*	**27.3%** (2.3)		*3*	**37.5%** (1.7)		*3*	**21.3%** (3)	
Duration of current episode, mean*(SD)*	*22*	**3 weeks** *(1.3)*		*8*	**2.9 weeks** *(1.1)*		*14*	**3 weeks** *(1.8)*	
Positive family history	*7*	**31.8%**		*2*	**25%**		*5*	**35%**	
Psychosocial strain	*3*	**13.6%**		*2*	**25%**		*1*	**7%**	
Outpatient	*15*	**68.2%**		*5*	**62.5%**		*10*	**71.4%**	
Inpatient	*7*	**31.8%**		*3*	**37.5%**		*4*	**28.6%**	

*S.D., Standard deviation; n, number of participants; BDI, Beck Depression inventory; HAM-D, Hamilton Depression scale; MADRS, Montgomery–Åsberg Depression Rating Scale Indicated are demographical facts, mean questionnaire scores as well as chronology and various clinical factors of all participants, as well as divided into subgroups of responders and non-responders. Positive family history refers to a first-degree relative with MDD. Psychosocial strain refers to exceptional events or stressful periods preceding symptoms. Out- and inpatient treatment refers to the time-period of the study only. There were no significant differences between the two groups concerning chronology and clinical factors*.

We found no significant differences between responders and non-responders in regards to gender (chi-square test: χ(1) = 2.424, *p* = 0.119), various clinical factors like previous psychiatric history, family history for depression, or treatment setting (see Table 1), or for scores of CTQ, EPI, ERQ, or mean picture ratings (for average values see Supplementary Table 1). The participants all received an SSRI, 20 received escitalopram in dosages between 5 and 20 mg, 1 participant received citalopram 20/40 mg, and 1 participant received sertraline 50 mg. Additionally, two patients (both non-responders) received mirtazapine as add-on.

No participants had received an antidepressant for treatment of the current episode prior to the study, two participants had lorazepam if needed until 1 week before the inclusion. There was no significant association between maximal dosage of respective antidepressant and therapy response [Spearman's correlation, *p* = 0.544, calculated with dose equivalent of escitalopram, (33)]. Additional to antidepressant medication, all participants had access to psychotherapeutic sessions (cognitive behavioral therapy), usually once weekly, but the exact modality was determined by treating clinician. Average values in the childhood trauma questionnaire (see Supplementary Table 1) were well within the range of what has been demonstrated in patients with major depressive disorder before (34).

Paired samples *t*-test revealed post-treatment BDI scores to be significantly lower than pre-treatment scores, this also applied to HAM-D, MADRS (see Supplementary Table 2). There was a significant association between higher pre-treatment BDI and higher change in BDI (Spearman's *r* = 0.436, sig. *p* = 0.042), which may be associated to the fact that antidepressants have been proven more efficient in moderate to severe depression (1). Because of this, we additionally did a correlation analysis (Spearman's correlation) to search for significant associations between pre-treatment BDI and the extracted beta weights in our regions of interest, in order uncover whether the differences in brain activity might just reflect more severe depression. Other than a significant inverse association between pre-treatment BDI and mean picture rating (Spearman's *r* = −0.63, sig. *p* = 0.002), correlation analysis of the various questionnaires with depression scores and extracted beta weights revealed no meaningful associations.

### fMRI Analysis Results

The whole brain correlation analysis with change in BDI was conducted for each contrast separately (Positive>neutral, negative>neutral, exp. negative>exp. neutral, and exp. unknown>exp. neutral).

#### Positive>Neutral

**Figure 2:**
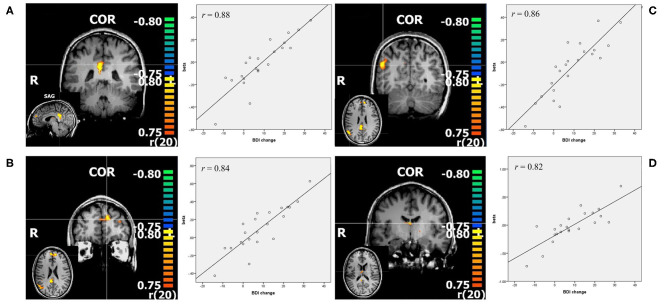
Resulting regions of correlation analysis with change in BDI in contrast positive>neutral. The color bars represent *r* values, *p* < 0.00005. **(A)** PCC: posterior cingulate cortex; **(B)** mFG: middle frontal gyrus; **(C)** mTG: middle temporal gyrus; **(D)** thalamus, all shown in coronal slice, with sagittal or transverse slice shown in small. Additionally scatterplots of the depicted regions beta values and change in BDI are shown, as well as *r* = Pearson's correlation coefficient.

For this contrast *p* was set at <0.00005, and the calculated min. cluster size was 10 voxels (Cluster Thresholding Estimator (CTE) Plugin, run with FWHM (Full width at half maximum) of 3.098 and 1,000 iterations). This revealed the bilateral posterior cingulate cortex (PCC), bilateral middle frontal gyrus (mFG), as well as the right middle temporal gyrus (mTG). With *r* values over 0.8 (Pearson's correlation, calculated by BrainvoyagerQX and verified by SPSS), these regions showed a strong correlation with change in BDI. Correlation of beta weights of those ROI's with pre-treatment BDI was not significant (See Figure 2, Table 2).

**Table 2:** Correlation analysis: activity in regions of interest significantly associated with change in BDI.

**Anatomic region**	**BA**	**Cluster size**	**x**	**y**	**z**	***r***	***p***	***r (BDI T1)***
**Positive** **>neutral**								
Posterior cingulate cortex R>L	23	2,356	6	−37	25	**0.8807**	0.000000	0.25
Middle frontal gyrus L>R	9	974	−9	50	25	**0.8360**	0.000001	0.29
Thalamus R		221	3	−10	16	**0.8173**	0.000003	0.15
Middle temporal gyrus R	39	1,697	48	−58	25	**0.8615**	0.000000	0.29
**Negative** **>neutral**								
Thalamus L		1,220	−18	−22	1	**0.6635**	0.000761	**0.54 (0.009)**
Posterior cingulate cortex L>R	29	1,294	−6	−46	10	**0.7064**	0.000239	0.33
Hippocampus R		976	30	−31	−8	**0.652**	0.001010	**0.49 (0.022)**
Anterior cingulate cortex R = L	32	1,104	12	32	28	**0.6626**	0.000779	0.13

*BA, Brodmann area; r, Pearson's correlation coefficient; p where significant, BDI T1, pre-treatment BDI. Activated regions according to the whole brain correlation analysis in the contrasts positive>neutral and negative>neutral. Indicated is the cluster size of each region in voxels, the Tailarach coordinates (x, y, z) of the peak activation of the cluster, and Pearson's correlation coefficient between change in BDI and beta values, as well as the corresponding p-value. The thalamic region in contrast positive>neutral corresponds to the medial dorsal nucleus, and in contrast negative>neutral to the ventral posterior lateral nucleus. The anterior cingulate cortex refers specifically to the pregenual anterior cingulate. The correlation coefficient r is printed in bold when significant*.

#### Negative > Neutral

**Figure 3:**
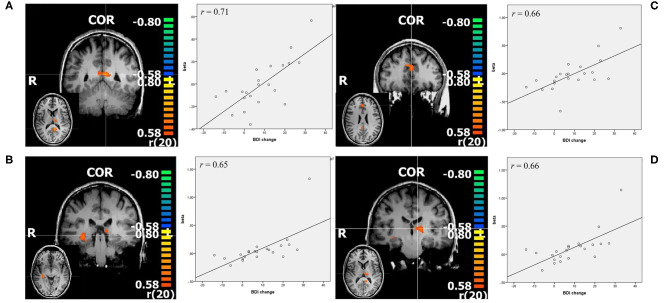
Resulting regions of correlation analysis with change in BDI in contrast negative>neutral. The color bars represent *r* values, *p* < 0.005. **(A)** PCC: posterior cingulate cortex; **(B)** right hippocampus; **(C)** ACC; pregenual anterior cingulate cortex; **(D)** left thalamus, all shown in coronal slice, with transverse slice shown in small. Additionally scatterplots of the depicted regions beta values and change in BDI are shown, as well as *r* = Pearson's correlation coefficient.

With *p* < 0.005 and a minimal cluster size of 32 (CTE Plugin, FWHM 2.345, 1,000 iterations) correlation analysis in this contrast revealed bilateral PCC, pregenual anterior cingulate cortex (ACC), left thalamus, and right hippocampus (see Figure 3, Table 2). While the beta weights from PCC and ACC did not correlate with pre-treatment BDI, those of right hippocampus and thalamus did.

#### exp. Negative >exp. Neutral

With a *p* < 0.005, and a minimal cluster size of 30 voxels there were no areas of interest.

#### exp. Positive >exp. Neutral

With a *p* < 0.005 there were no areas of interest.

#### exp. Unknown > exp. Neutral

With a *p* < 0.005 and a minimal cluster size of 19 (CTE Plugin, FWHM 1.899, 1,000 iterations) there was significant activation in an area including parts of the right inferior parietal lobule. Due to the expansion of the area outside gray matter, we considered this an artifact.

#### Inverse Pattern Between Responders and Non-responders

**Figure 4:**
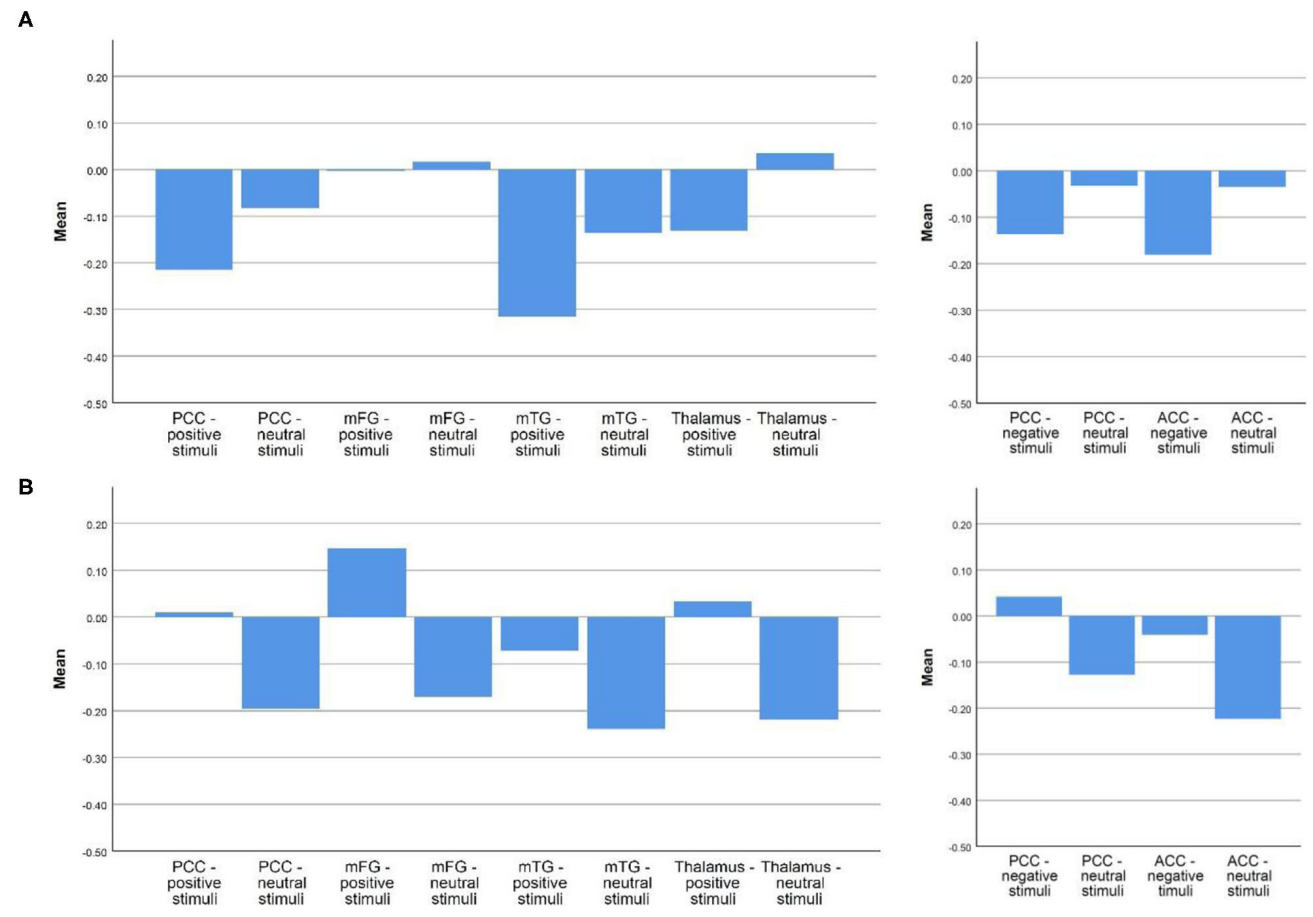
Mean betas in responders and non-responders in each region of interest during the perception of emotional vs. neutral stimuli. **(A)** Non-responder; **(B)** Responder. Depicted are the mean betas of responders and non-responders in our regions of interest in the respective emotional and neutral condition, the two graphs on the left site concern the regions more active in responders during the perception of positive stimuli, the two graphs on the right the ones more active in responders during the perception of negative stimuli.

When examining activity in each condition on its own (instead of the contrast), there was a difference between responders and non-responders, which was seen to some degree in all the above-mentioned regions. Responders were characterized by higher activation during emotional stimuli compared to neutral stimuli, as well as compared to non-responders perceiving positive stimuli. Non-responders demonstrated attenuated activation during the perception of emotional stimuli compared to neutral stimuli. For visualization, see Figure 4.

## Discussion

We examined whether neurobiological markers revealed from fMRI during emotion perception are associated with treatment response in depression in order to determine potential treatment response predictors. We found the following differences between responders and non-responders:

(i) During the perception of *negative s*timuli, responders were characterized by higher activation in bilateral pregenual ACC (BA 32), on the border to the anterior dorsal ACC, bilateral PCC (BA 29), as well as ventral posterior lateral nucleus of thalamus and right hippocampus. During the perception of *positive* stimuli, responders exhibited higher activation in bilateral PCC (BA 23), bilateral mFG (BA 9, medial prefrontal cortex), right medial dorsal nucleus of thalamus, and right mTG (BA 39), corresponding to the angular gyrus.
(ii) There was an interesting difference of the relation between activation during emotional vs. neutral stimuli in the two groups. While responders generally showed more activation during emotional than during neutral conditions (emotional >neutral), non-responders demonstrated an attenuation of activation during emotional conditions compared to neutral ones (neutral >emotional). When subtracting betas during neutral conditions from betas measured in emotional conditions this results in inverse algebraic signs.

Activity in ACC during emotional stimuli predicting treatment response is in line with several previous studies (35–38). Confirming this not only as a marker, but also as an antidepressant target in MDD, (39) found decreased activation in the ACC during a negative emotion task after treatment with a SSRI. However, emotional task designs varied, our task design combined pictures which might reflect anger, induce sadness, or provoke fear all as negative stimuli. In their treatment response prediction study with a face matching task (11) found a significant positive treatment response prediction for angry faces, and an inverse association with fearful faces.

The association between higher baseline anterior cingulate activity and better treatment response has been found by a more diverse set of studies, and has been meta-analytically confirmed (13, 40). Better treatment response has been demonstrated also for greater gray-matter volume of ACC (35). Dunlop et al. (41) showed higher connectivity between the subcallosal cingulum and various regions to predict treatment response to either antidepressant therapy or CBT, and several other studies showed the ACC as a treatment predictor for Behavioral Activation Therapy (42, 43).

Traditionally, the ACC has been divided into a dorsal—cognitive part, and a ventral—emotional part (44), but this classification has been challenged in recent years. Etkin et al. (45) proposes both sections to be involved in emotion processing, but with the dorsal regions playing a role in evaluation and expression of negative affect, and the ventral regions (subgenual and pregenual ACC) having a regulatory role by inhibiting the emotional response via the limbic system. Our results support the notion that, additionally to resting state, activity in the ACC during perception of negative stimuli is a marker of treatment-response to antidepressants, possibly through better regulatory control of emotional networks.

Activity in thalamus and right hippocampus during perception of negative stimuli was also associated with treatment response, but in contrast to the other regions of interest also significantly correlated with pre-treatment BDI. Thus, this might also reflect a biomarker of more severe depression, instead of a treatment response predictor. Functional and structural changes of Hippocampus in depression have been well-established (8, 9), but plasticity under treatment has also been demonstrated by Arnone et al. (46) where hippocampal gray matter reduction reverted under successful treatment with SSRI (citalopram), pointing it out as biomarker for early intervention.

However, activity in PCC was one of the strongest predictor of treatment response in our data, which has been associated to pharmacological treatment response before (47–49). PCC and ACC have both been assigned to the default mode network (DMN) (50), which refers to several regions that have been demonstrated active during resting state, independent from external stimuli (18). The DMN has also been shown to be involved with self-referential processing, e.g., relating (external) information to the self (51) and retrieval of memories (52, 53). While the DMN was initially thought to deactivate when performing a cognitive task, this opinion has been challenged, and its function is thought to go beyond passive resting state. It has been divided into several distinct subsystems, with a *core system* (comprising of PCC, angular gyrus and amPFC including the cingulate cortex), a *dorsal medial subsystem*, and a *medial temporal subsystem* (19).

Many of our found regions match to this network. PCC, mPFC including ACC as well as mTG (corresponding to the angular gyrus) could be attributed to the *core system*.

The core system is thought to be extensively connected to the rest of the DMN, and mainly involved in self-referential processing of thoughts/information and integration with autobiographical memories or knowledge, possibly constructing a “personal meaning” from internal or external input (19). The *dorsal medial subsystem* is involved rather in metacognitive processes like mentalizing, while the *medial temporal subsystem* plays a role in retrieving autobiographical memories and simulating future scenarios. While the thalamus is itself not described as a part of the DMN, patients with MDD seem to have significantly higher connectivity to the DMN compared to controls (54, 55). This might explain the similarly high activity of thalamus and PCC in our responders.

Disruptions of the DMN have been demonstrated for various psychiatric disorders. For example, negatively biased self-generated thoughts and heightened activity of the DMN have been associated with depression (20, 21, 54, 56–58), as well as maladaptive rumination (55). Maladaptive rumination (“brooding”) has been associated with higher depressive symptoms in the later course of the disease (59). Our results of responders exhibiting higher activity in core areas of the DMN might be an expression of rumination and more active self-referential processes during the perception of both positive and negative emotional stimuli.

Altered activity in cortical midline structures, corresponding to areas of the DMN has also been viewed as a treatment target [see (17) for a review]. For example, Di Simplicio et al. (60) found that citalopram attenuated neural response in mPFC and ACC during negative word categorization in a self-referential task in participants at risk for depression. Posner et al. (61) found that duloxetine, and not placebo normalized heightened DMN connectivity in resting state in patients with dysthymic disorder. Arnone et al. (62) showed citalopram to increase static connectivity between areas of the DMN as opposed to placebo. Fu et al. (63) found SNRI (duloxetine) to significantly alter DMN connectivity in response to negative attentional biases. In a longitudinal pharmacological fMRI study, (64) demonstrated enhanced de-activation of the amPFC—as mediator of the DMN—under escitalopram being associated with recovery. Nejad et al. (17) also supported the notion of antidepressant treatment leading to diminished neural response to negative self-referential content.

Our finding (ii), the inverse pattern of activation seen between responders and non-responders when examining each condition on its own illustrates the differences between the two groups. Responder exhibited higher activation during perception of emotional stimuli (both positive and negative) than neutral stimuli, and non-responder higher activity during perception of neutral stimuli than emotional ones (see Figure 4).

Higher activity in DMN regions during perception of negative stimuli in patients with MDD is a known alteration (58). In contrast, healthy controls showed no significant differences between negative and neutral stimuli (65). We propose that the higher activity of our responders during negative stimuli compared to neutral ones reflects stronger self-referential activity in DMN regions and therefore a negative cognitive bias, which might be remedied by antidepressant therapy (17, 22, 66).

On the other hand, the pattern in our non-responders with attenuated activation during perception of positive stimuli compared to neutral stimuli might reflect less positive self-referential processing/anhedonia, resulting in higher resistance to therapy. Kumari et al. (67) found participants with treatment resistant depression to exhibit lower activation in several regions including ACC, Thalamus, and PCC during perception of positive stimuli, which matches the pattern seen in our non-responders. In comparison, healthy controls were shown to exhibit higher activation during the perception of happy faces compared to neutral faces in DMN regions (65).

Our finding (ii) is additionally interesting in regards to treatment prediction on an individual level. When subtracting betas of regions of interest during neutral conditions from betas measured in emotional conditions this results in inverse algebraic signs in responders vs. non-responders. Many previous studies demonstrated differences in activation between responders and non-responders, but the difficulty is to define a cut-off, which can be used for treatment prediction when looking at a single patient. However, the inverse relation that our responders/non-responders showed, doesn't need a cut-off, since it clearly divides them into two groups.

### Strengths and Limitations

Regarding recruiting and evaluation of psychometric data, we see the small number of participants with an imbalance between responders (*n* = 8) and non-responders (*n* = 14), and an imbalance between genders in the total sample (9 male and 13 female) as limitations. Due to the nature of this study, we also did not have a control group, since it was not supposed to be randomized pharmacological trial, but rather intended to recruit patients within a short time window—after the diagnosis of an episode of MDD had been confirmed but before the beginning with antidepressant medication. The lack of a control group receiving placebo is a widespread phenomenon in response prediction studies (40), most likely because of the ethical challenge this implies. With antidepressants being more effective than placebo (2) it is difficult to justify withholding more effective treatment. But as with any treatment, a certain placebo effect is to be expected with antidepressants (68), and the differences in brain activitiy in our responders might also just point out the participants which would also get better with placebo (69). However, since antidepressants seem to be even more efficient than placebo in severe depression (1), and our participants showed more BDI reduction dependent on the severity of depression, we assume this to mostly due to treatment with an SSRI.

Another limiting factor due to our study design is the use of only one neuroimaging modality (4), however, in clinical practice a simple application for treatment response prediction (TRP) is needed with a useful approximate result.

As advantage, we see the fact that one single psychiatrist evaluated all the participant's psychometric data, which excludes a bias due to different examiners.

Regarding fMRI data acquisition, it was not possible to make sure that participants paid attention during the complete scan due to the nature of our (passive) experimentation setup, which might falsify results. However, we performed exploratory analysis of the activation in the visual cortex during the different conditions, which showed no relevant differences between the group of responders and non-responders. Regarding (pharmacological) treatment, we didn't limit the antidepressant medication to one specific substance, so that one participant received a different SSRI (sertraline), and one received citalopram, the precursor of escitalopram. In addition to psychopharmacological treatment, all participants were seen regularly by a psychiatrist and had access to some form of psychotherapy. Therefore, it is not possible to specifically attribute treatment success to the SSRI. As strength of this study we see the analysis of both negative and positive conditions in regions of interest, while most previous studies focused on negative contrasts, furthermore the experimental design allowed analysis of the neutral condition as well.

## Conclusion

Our results support the findings that functional brain activity can act as treatment predictor. Most of our regions of interest matched core regions of the DMN, corresponding to previous findings of altered DMN activity in depression, which may reflect increased (negative) self-referential processes and rumination. This coexists with diminished ability of regulation, and the significant differences between responders and non-responders support the notion of altered neural activity in the DMN being a target of the antidepressant mode of action of SSRI, and/or predicting treatment response. Our result of non-responders exhibiting attenuated activity to positive and negative emotional stimuli compared to neutral ones, with responders demonstrating the opposite pattern are especially interesting in regards to finding a treatment-response predictor which could be applied at the individual level, and warrants further investigation.

## Data Availability Statement

The datasets generated for this study are available on request to the corresponding author.

## Ethics Statement

This study involving human participants was reviewed and approved by the Cantonal Ethics Committee of Zurich. The participants provided their written informed consent to participate in this study.

## Author Contributions

AP, UH, AB, and BB contributed conception and design of the study. AP was responsible for most of the fMRI and psychometric data acquisition. BB performed data organization and statistical analysis and wrote the first draft of the manuscript with continued input of AP, who also wrote sections of the manuscript. All authors contributed to manuscript revision, read, and approved the submitted version.

## Conflict of Interest

The authors declare that the research was conducted in the absence of any commercial or financial relationships that could be construed as a potential conflict of interest.
