# Supplementary material for: SSRI Treatment Response Prediction in Depression Based on Brain Activation by Emotional Stimuli
Source: Front Psychiatry. 2020 Nov 13;11:538393. doi: 10.3389/fpsyt.2020.538393 (PMC7691246; doi:10.3389/fpsyt.2020.538393)
Supplement: Supplementary file 1 [file Data_Sheet_1.PDF]

Supplementary Material

Tables

Table S1
Descriptive Statistics
S.D.: Standard deviation, BDI: Beck Depression inventory, HAM-D: Hamilton Depression scale, MADRS: Montgomery–Åsberg Depression Rating Scale, EPI: Eysenck Personality Inventory, ERQ: Emotion Regulation Questionnaire, CTQ: Childhood Trauma Questionnaire
Indicated are demographical facts as well as mean questionnaire scores of all participants, as well as divided into subgroups of responders and non-responders.
There were no significant differences between responders and non-responders regarding the results of EPI, ERQ, or CTQ.
[1] 5-9 none to minimal, 10-14 low to moderate, 15-17 moderate to severe, >=18 severe to extreme
[2]>=5 none to minimal, 6-7 low to moderate, 8-12 moderate to severe, >=13 severe to extreme
[3] 5-7 none to minimal, 8-9 low to moderate, 10-12 moderate to severe, >=13 severe to extreme
[4] 5-8 none to minimal, 9-12 low to moderate, 13-15 moderate to severe, >=16 severe to extreme

	All			Responder			Non-responder		
	n		*S.D.*	*n*		*S.D.*	*n*		*S.D.*
BDI T1	*22*	**27.0**	*8.0*	*8*	**30.0**	*7.3*	*14*	**25.2**	*8.2*
BDI T6	*22*	**18.4**	*12.0*	*8*	**8.5**	*4.3*	*14*	**24.1**	*11.2*
HAM-D T1	*22*	**25.5**	*6.1*	*8*	**26.3**	*4.5*	*14*	**25**	*6.9*
HAM-D T6	*22*	**18.1**	*10.1*	*8*	**8.3**	*5.7*	*14*	**23.6**	*9.1*
MADRS T1	*22*	**28.6**	*7.4*	*8*	**30.5**	*7.3*	*14*	**27.6**	*7.6*
MADRS T6	*21*	**20.4**	*10.1*	*7*	**9.9**	*3.7*	*14*	**25.7**	*7.7*
EPI									
Neuroticism	*22*	**17.4**	*8.7*	*8*	**19.3**	*13.6*	*14*	**16.3**	*4.4*
Extraversion	*22*	**10.2**	*4.7*	*8*	**12.4**	*5.9*	*14*	**9.0**	*3.4*
Lying	*22*	**4.8**	*1.8*	*8*	**5.0**	*2.4*	*14*	**4.7**	*1.4*
ERQ									
Reappraisal	*21*	**3.8**	*1.1*	*7*	**4.0**	*1.2*	*14*	**3.7**	*1.1*
Suppression	*21*	**3.5**	*1.1*	*7*	**2.9**	*0.7*	*14*	**3.7**	*1.2*
CTQ									
Emotional neglect[1]	*22*	**13**	*4.9*	*8*	**12**	*4.4*	*14*	**13.6**	*5.3*
Sexual abuse[2]	*22*	**6.9**	*3.5*	*8*	**7.4**	*3.2*	*14*	**6.6**	*3.7*
Physical abuse[3]	*22*	**7**	*2.7*	*8*	**7.5**	*4.0*	*14*	**6.6**	*1.7*
Emotional abuse[4]	*22*	**9.8**	*4.7*	*8*	**9.3**	*5.6*	*14*	**10.1**	*4.4*
Physical neglect[3]	*22*	**8.3**	*2.9*	*8*	**7.9**	*3.0*	*14*	**8.6**	*2.9*
Mean picture ratings									
All	*21*	**5.0**	*0.4*	*8*	**4.9**	*0.4*	*13*	**5.1**	*0.4*
Positive	*21*	**7.6**	*1.2*	*8*	**7.2**	*1.7*	*13*	**7.9**	*0.7*
Negative	*21*	**2.2**	*0.7*	*8*	**2.4**	*0.9*	*13*	**2.1**	*0.7*

Table S2

Paired sample T-Test for depression

T1: pre-treatment, T6: post-treatment, S.D.: Standard deviation, Std.: standard, C.I.: Confidence Interval, *t*:*t*-value, df: degrees of freedom, Sig.: significance (2-tailed), BDI: Beck Depression inventory, HAM-D: Hamilton Depression scale, MADRS: Montgomery–Åsberg Depression Rating Scale,

Presented are the results of paired sample T-Tests for pre- and posttreatment depression first for the whole sample, demonstrating a significant reduction of scores after treatment, as well as for the group of responders and non-responders on their own.

	Mean	S.D.	Std. Error Mean	95% C.I. of the Difference		t	df	Sig.
				Lower	Upper			
BDI T1 – BDI T6	8.545	12.031	2.565	3.211	13.880	3.332	21	.003
HAM-D T1 – HAM-D T6	7.409	10.340	2.205	2.824	11.994	3.361	21	.003
MADRS T1 – MADRS T6	8.333	11.297	2.465	3.191	13.476	3.380	20	.003
**Responders**								
BDI T1 – BDI T6	21.500	6.414	2.268	16.138	26.862	9.481	7	.000
HAM-D T1 – HAM-D T6	18.000	4.957	1.753	13.856	22.144	10.271	7	.000
MADRS T1 – MADRS T6	21.286	7.296	2.758	14.538	28.034	7.718	6	.000
**Non-responders**								
BDI T1 – BDI T6	1.143	7.026	1.878	-2.914	5.199	0.609	13	.553
HAM-D T1 – HAM-D T6	1.357	7.143	1.909	-2.767	5.481	0.711	13	.490
MADRS T1 – MADRS T6	1.857	6.024	1.610	-1.621	5.335	1.154	13	.269
